# Efficacy of *Pythium oligandrum* on improvement of lucerne yield, root development and disease score under field conditions

**DOI:** 10.3389/fpls.2022.1045225

**Published:** 2022-12-06

**Authors:** Martin Pisarčik, Josef Hakl, Ondřej Szabó, Pavel Nerušil

**Affiliations:** ^1^ Department of Agroecology and Crop Production, Czech University of Life Sciences Prague, Prague, Czechia; ^2^ Research Station of Grassland Ecosystems Jevíčko, Crop Research Institute, Jevíčko, Czechia

**Keywords:** alfalfa, Medicago sativa, plant growth promotion, Polyversum, dry conditions

## Abstract

**Introduction:**

Biological control of root diseases of lucerne (Medicago sativa L.) has potential benefits for stand performance but this remains unsupported by evidence from practical field studies.

**Methods:**

In field experiments at three sites our objectives were to determine the effect of Pythium oligandrum, as spring, autumn and intensive regime treatments on (i) lucerne plant density and root traits development, and (ii) forage yield and forage traits. Lucerne stands were managed under two or three treatments: non-treated control and P. oligandrum applied at two intensities of application under four-cut utilization.

**Results and discussion:**

Under relatively dry conditions (annual mean 10°C and <500 mm precipitation) lucerne dry matter yield was significantly reduced by 6%, which could be related to mechanisms of inappropriate stimulation and disturbance of the balance between auxins and ethylene. Under annual precipitation of >500 mm, positive impacts on stand height or fine root mass were observed for the autumn and intensive treatments where positive root response was visible only in alluvial soil. However, these changes did not result in higher yield and probably more applications per year will be needed for significant forage yield improvement. This study highlights the limits of field-scale biological control in which the potential of P. oligandrum for lucerne productivity improvement was realised only under a humid environment or deep alluvial soils, where higher root disease infestation may also be expected.

## Introduction

Biological control of pests and diseases of field crops has become of increasing importance in recent years in line with the current emphasis on improved sustainability of crop production and reduced use of chemical pesticides in agriculture. Increasing implementation of biological approaches, including biological control, biopesticides, biostimulants and pheromones, is a high priority for sustainable agriculture leaders and farmers (Baker et al., 2020). Among potential biological control agents, beneficial fungi have received significant attention worldwide due to their remarkable antagonistic properties against plant pathogens and numerous successful applications have been reported (Ghorbanpour et al., 2018). Several studies on the use of fungi as biological control agents have identified multiple mechanisms by which fungal agents, such as *Pythium* (Gerbore et al., 2014) or *Trichoderma* (Ghorbanpour et al., 2018) may protect plants from pathogens, as well as showing the limits for their effective utilization in affecting soil-borne diseases (Campbell, 1994). Understanding the mechanisms for fungal control of pathogens is crucial for the development of effective biocontrol strategies against plant diseases, and there are further requirements affecting their use such as the ability to produce liquid cultures for easy application and ensuring an adequate persistence of the fungal agent in the environment (O’Brien, 2017). In addition to the biocontrol properties of *Trichoderma*, as highlighted by Ghorbanpour et al. (2018), *Pythium oligandrum* has been investigated for its potential as a biological protection agent for several crops over the last two decades (Gerbore et al., 2014), either alone or in combination with bacterial strains (Ouhaibi-Ben et al., 2021). Benhamou et al. (2012) summarized that *P. oligandrum* is a mycoparasitic oomycete, and has the ability to colonize the rhizosphere and root systems of many crop plants, directly attacking soil-borne fungal pathogens, promoting plant growth, and increasing crop protection against fungal disease *via* the activation of the plant’s immune system. Examples of the efficacy of *P. oligandrum* against the plant pathogens have been reported for *P. ultimum, P. aphanidermatum, Fusarium oxysporum*, *Verticillium albo-atrum, Rhizoctonia solani* and *Physperma solani* (Benhamou et al., 1999, 2012). Bělonožníková et al. (2020) described differences among 12 diverse strains of *P. oligandrum* with regard to their properties and effect on plants, and identified strain M1 with positive influence on plant fitness.

Forage legumes such as lucerne (*Medicago sativa* L.) or red clover (*Trifolium pratense* L.) comprise an important group of field crops that are susceptible to root diseases which can seriously limit their survival and yield through reduction of plant density and productivity over successive years (Sedman et al., 2007). Although a number of studies have been conducted over several decades, no effective fungicidal control of these root diseases has yet been established or approved. This is partly because fungicidal control by chemical fungicides is thought to be ineffective in providing complete disease control (McKenna et al., 2018) and several studies have reported reduced crop growth efficiency, especially in terms of forage yield improvement (Larkin et al., 1996; Hwang et al., 2002; Gray and Koch, 2004). Although some studies have demonstrated positive effects of fungicides on disease protection of forage legumes (Leath et al., 1973; Larkin et al., 1995), this was usually followed by negative effects on crop growth (Jenkyn, 1975; Nan et al., 1991) and/or adverse effects on non-target microorganisms such as mycorrhizal fungi (Yang et al., 2011) and N fixation, where inhibition of molecular signalling between rhizobia and the host legume plants has been observed (Ahemad and Khan, 2013). Abbas et al. (2022) suggested also suitable combinations of fungicide with biological control agents reducing environment pollution. The absence of an approved fungicide protection treatment has meant that plant breeding for disease resistance (Riday, 2010) and/or suitable cultivation management (Gray and Koch, 2004) remain as the key disease-control strategy for lucerne or red clover producers (2020; Pisarčik et al., 2019).

Among biological control methods, some positive effects for lucerne were reported after *Streptomyces* (Jones and Samac, 1996; Xiao et al., 2002) or *Bacillus cereus* application (Handelsman et al., 1990), both of which reduced lucerne seedling damping-off during stand establishment. Morsy et al. (2011) observed a positive response of lucerne to application of *Bacillus megatherium* or *Trichoderma album*, although neither was as effective as abiotic agents in their study. Öhberg and Bang (2010) confirmed there was a positive yield effect of application of *Conithirium minitans* prior to seeding, when red clover plants were infected with *Sclerotinium trifoliorum*. Despite some positive examples of biological control, Pisarčik et al. (2019) found that biological control under field conditions may not always give beneficial outcomes, in contrast to the results of laboratory or greenhouse experiments, because of complicated interactions between plant, organism/preparation, and environment resulting in general unstable controllling effects from year to year. Although there has been intensive research in biological protection of diseases under controlled conditions, there still remains a lack of field-based studies to provide evidence supporting the economic efficiency of biological control for particular crops, especially for the successive harvest years of perennial forage crops. Among the *Fabaceae* crops, the effectiveness of *P. oligandrum* has been documented mostly for grain legumes such as soybean (You et al., 2019) or pea (Nikolova et al., 2015) and only a few studies have been reported for forage legumes. The *Fusarium* and *Verticilium* generally represent the main root pathogens of lucerne (Miller-Garvin and Viands, 1994) and were also identified as the key genera of lucerne root pathogens in central Europe (Hakl et al., 2017). Efficacy of *P. oligandrum* (strain M1) against *Fusarium* (*F. avenaceum*, *F. oxysporum*, *F. culmorum*, and *F. solani*) in red clover stands has been presented (2021; Pisarčik et al., 2020)

Positive effects on forage yield have been reported in two independent experiments with red clover under treatments with intensive application of *P. oligandrum* but this improved yield was associated more with plant growth stimulation than from a direct protection against *Fusarium* (2021; Pisarčik et al., 2020). In lucerne, spring application of *P. oligandrum* was found to provide zero effect on yield and it even increased the proportion of infected plants in the last year of the field experiment (Pisarčik et al., 2019). For vetch (*Vicia sativa* L.), significantly lower effects of biological control than with conventional chemical control have been reported (Georgieva et al., 2020). Previous research has also shown a significant relationship between lucerne root morphology and infection of root diseases (Larkin et al., 1995; Hakl et al., 2017) and therefore there is a need for evaluation of disease score together with root traits and forage yield.

Optimization of the number of applications and dates of application is needed to ensure economic practice in crop protection (Pisarčik et al., 2021). This is especially difficult for perennial forage crops that are utilized over multiple-year growing seasons covering the seeding year and two or three subsequent harvest years. The results with a single spring application of *P. oligandrum* in lucerne have not provided positive yield response at the one location investigated so far (Pisarčik et al., 2019). For red clover some benefits under an intensive or autumn application regime, in contrast to spring application, have been reported in terms of improved red clover yield and/or root traits (Pisarčik et al., 2021). These previous outcomes for red clover highlights potential of *P. oligandrum* for forage legumes and gives further encouragement to extend the biological control research with treatments including variable timing of applications to lucerne. Testing effective lucerne protection would have an innovative benefit as no such practical method has yet been recommended for field conditions. Therefore, in the work reported here, we have combined results obtained from three field experiments that investigated different timing and intensity of lucerne biological control of root diseases. Our aims were to investigate the effect of spring, autumn and intensive regimes of *P. oligandrum* application to lucerne on (i) plant density and root traits development, and (ii) on forage yield and forage traits in a four-year field experiment on different sites. An additional aim was to evaluate the relationship between lucerne fine root mass, other root morphology traits and plant root disease score. These comprehensive evaluations from different locations could be valuable for better understanding of the simultaneous effects of tested treatments on the improvement of lucerne stand performance and provided outcomes can be practically applied in the similar field conditions.

## Materials and methods

### Field experiments

Field plot experiments with lucerne were conducted at three sites in the Czech Republic during 2016 - 2020. Site 1 (Červený Újezd, elevation 410 m a.s.l.) is characterized by clay-loam Haplic Luvisol with a shallow soil profile, long-term mean annual temperature of 8.4°C and cumulative annual rainfall of 502 mm. Site 2 (Jevíčko, elevation 350 m a.s.l.) is located on a river floodplain with a deep loam Haplic Luvisol and high soil humidity. Site 3 (Drválovice, elevation 460 m a.s.l.) is characterized by loamy-sandy Cambisol. Sites 2 and 3 are close to each other and share data from the same meteorological station: long-term mean annual temperature of 7.4°C and cumulative annual rainfall of 545 mm. All characteristics of sites are summarized in **Table 1**.In all three experiments, lucerne varieties Oslava (at Sites 1 and 2) and Pálava (at Site 3) were established in spring as monocultures with a sowing rate of 700 viable seeds m^-2^ and row seeding at a between-row distance of 12.5 cm. Each experiment consisted of a non-treated control and one or two Polyversum treatments, and was arranged in a completely randomized block design with four replicate blocks. Plot size was 7.2 × 2.5 m with a harvested area of 10 m^2^. The preparation of Polyversum (Biopreparáty spol. s r.o., Czech Republic) contained 1 000 000 active oospores of *Pythium oligandrum* M1 per gram. The Polyversum was applied once each year (in spring or autumn) with additional applications per year for the intensive treatment. In the seeding year, Polyversum treatments were applied one to three times (6 weeks after stand establishment, after an initial mowing of the new sward at 5 cm for which the cut herbage mass was not recorded), and also after an autumn cut, depending on intensity of application. Timing of Polyversum applications in post-seeding years corresponded with a four-cut schedule where the number of applications for the intensive treatment varied from two at Site 2 (after first and autumn cut) to five at Site 1 (in spring and after each cut). The single applications of Polyversum were made after first cut (spring treatment) or after the autumn cut (autumn treatment). The timing of all applications across years and experiments is described in **Table 2**. Polyversum was always activated one hour before application, and the rate was 100 g ha^-1^ applied in 300 litres of water, using a backpack sprayer (Cooper Pegler 15 EVO) with a working pressure of 3 bar, always in humid weather without intensive sunshine.

**Table 1:** Description of localities, annual temperature mean and cumulated precipitation.

Locality		Červený Újezd (Site 1)	Jevíčko (Site 2)	Drválovice (Site 3)
Elevation (m a.s.l.)		410	350	460
GPS coordinate		50.07207 N 14.17136 E	49.62904 N 16.72815 E	49.56745 N 16.65041E
Soil texture and type		clay-loam Haplic Luvisol	deep loam Haplic Luvisol	loamy-sandy Cambisol
Temperature (°C)	2016	9.6 (+1.2)*	–	
	2017	9.5 (+1.1)	8.4 (+1.0)	
	2018	10.4 (+2.0)	9.6 (+2.2)	
	2019	10.4 (+2.0)	9.4 (+1.8)	
	2020	–	9.1 (+1.5)	
Precipitation (mm)	2016	475 (-27)*	–	
	2017	492 (-10)	539 (-6)	
	2018	334 (-168)	401 (-144)	
	2019	479 (-23)	574 (+30)	
	2020	–	722 (+178)	

*difference to long-term mean (1981 - 2010).

**Table 2:** Description of treatments, application dates of Polyversum at a dose rate of 100 g ha^-1^ in Polyversum treatments together with root sampling area and sampling dates, and forage harvest dates for all treatments in the years of the study.

Locality		2016	2017	2018	2019	2020
	Treatments	Application, sampling and harvest dates				
Červený Újezd (Site 1)	Autumn	6 Jun, 28 Oct	26 Oct	9 Oct	9 Oct	–
	Nr. of app.	2	1	1	1	–
	Intensive	6 Jun, 27 July, 28 Oct	11 Apr, 29 May, 7 Jul, 28 Aug, 26 Oct	13 Apr, 17 May, 21 Jun, 14 Aug, 9 Oct	12 Apr, 24 May, 9 Jul, 27 Aug, 9 Oct	–
	Nr. of app.	3	5	5	5	–
	Root sampling Area (cm)	2 Nov 25 x 12.5	14 Nov 50 x 12.5	16 Oct 50 x 12.5	29 Oct 50 x 12.5	–
	Forage harvest dates	20 Oct	18 May, 28 Jun, 17 Aug, 19 Oct	10 May, 14 Jun, 31 Jul, 9 Oct	16 May, 27 Jun, 12 Aug, 3 Oct	–
Jevíčko (Site 2)	Spring	–	3 Jun	25 May	11 Jun	–
	Nr. of app.	–	1	1	1	–
	Intensive	–	3 Jun 3 Nov	25 May, 15 Oct	11 Jun, 15 Oct	–
	Nr. of app.	–	2	2	2	–
	Root sampling Area (cm)	–	10 Nov 25 x 12.5	1 Nov 50 x 12.5	1 Nov 50 x 12.5	–
	Forage harvest dates		20 Jun, 31 Jul, 19 Sep	4 May, 11 Jun, 19 Jul, 20 Sep	17 May, 24 Jun, 9 Aug, 24 Sep	–
Drválovice (Site 3)	Spring/Autumn	–	23 May	15 May	15 Oct	12 Oct
	Nr. of app.	–	1	1	1	1
	Root sampling Area (cm)	–	14 Nov 50 x 12.5	1 Nov 50 x 12.5	12 Nov 50 x 12.5	18 Nov 100 x12.5
	Forage harvest dates	–	11 May, 16 Jun, 19Jul, 24 Aug, 11 Oct	4 May, 1 Jun, 19 Jul, 29 Aug, 9 Oct	16 May, 25 Jun, 12 Aug, 24 Sep	18 May, 22 Jun, 14 Aug, 25 Sep

### Lucerne root morphology and root disease score

In each plot of all three of the experimental sites, lucerne plants were dug each autumn to a depth of about 20 - 25 cm. The sampling area was increased over time to provide a similar number of plants under the natural decrease of plant density. Sampling dates and size of the root sampling areas are presented in **Table 2**. Lucerne plant density (PD, plants m^-2^) was calculated from the number of plants per sample and size of the root sampling area. For each plant, the tap-root diameter below the crown (TD, mm) and lateral root number per plant tap-root (LRN, when diameter larger than 1 mm) were measured. Presence of fine root mass (FRM, when less than 1 mm) was estimated subjectively on a scale of 1 to 5 with scores of 1, 3, and 5 indicating none, moderate and many fine roots, respectively. Washed root samples were oven-dried at 60°C for 48 h and total root dry matter (RDM, g m^-2^) was calculated based on the size of the sampling area. Plant root disease score (PRDS) was determined subjectively and based on discoloration on a cross-cut of the tap-root. The disease scoring followed Hakl et al. (2017): 0 = healthy plant, no discoloration in the root; living plants with root discoloration were scored from 1 to 6 with a score of 1 = 1 to 5%, 2 = 5 - 20%, 3 = 20 - 40%, 4 = 40 - 60%, 5 = 60 - 80%, 6 = 80 - 95% of the area of the tap-root cross-cut; 7 = dead plant. Ratio of infected plants (IP) was calculated from proportion of healthy plants and PRDS is reported as mean of infected plants between treatments or years. At stand level, the percentage of branch-rooted plants (root system with lateral roots developing from the tap root; RB) was calculated per sample. The root potential index (RPI) integrating TD and plant density was calculated according to equation proposed by Hakl et al. (2017). For precise detection of root pathogens, root samples (2 x 5 plants per plot) were taken in the autumn of the final year of the experiments. Taproots of five plants were combined, frozen and lyophilized. Quantitative real-time PCR (qPCR) were used for detection of key pathogens in the given environment, i.e. *Fusarium oxysporum*, *F. avenaceum* and *Verticillium albo-atrum*, separately in each sample. Primers were based on known sequences for *F. avenaceum* (Waalwijk et al., 2004), *F. oxysporum* (Haegi et al., 2013) and *Verticillium albo-atrum* (Maurer et al., 2013). All qPCR reactions were set in the Hard-Shell^®^ 96-Well PCR plates (BioRad) in a total volume of 10 µl by mixing 5 µl of gb Easy PCR Master Mix (twofold concentrated, Generi Biotech, Hradec Králové, Czech Republic), 1 µl of each of the qPCR assay mixture (tenfold concentrated), and 3 µl of the target DNA. PCR plate was placed into the heating block of the CFX Connect Real-Time PCR Detection System (BioRad) operated using CFX ManagerTM Software (ver. 3.0, BioRad). The cycling parameters were: 95°C for 3 min (initial denaturation) followed by 50 cycles of 95°C for 10 s (denaturation), and 50°C for 30 s (annealing plus extension). Fluorescence of FAM (λex = 495 nm, λem = 520 nm) and HEX (λex = 535 nm, λem = 556 nm) was monitored during every PCR cycle, after the extension step. The absolute DNA concentration was calculated using the linearized cloned target sequence standards (Generi Biotech, Hradec Králové, Czech Republic).

### Harvest management, sampling and measurement of forage structure traits and yield

In the seeding year, 2016, an initial unrecorded cut made in mid-summer was followed by an autumn harvest (at Sites 1 and 3) whilst three cuts were taken at Site 2. In the post-seeding years a four-cut management was applied at Sites 1 and 2, but at Site 3 there were five cuts per year taken in the first two years (2017 and 2018) and a four-cut schedule in 2019 and 2020. All dates of cutting are summarized in **Table 2**. Before each harvest in the post-seeding years, the compressed stand height (CH, cm) was measured using a metal rising plate meter with disc diameter 0.3 m, area 0.07 m^2^ and weight 0.2 kg, and six measurements per plot were taken according to Hakl et al. (2012). Fresh matter yield was assessed by harvesting 10 m^2^ in the centre of each plot, to a residual height of 5 cm, using a mower (MF-70, Agrostroj Jičín, Czech Republic) with a working width of 1.4 m. Fresh forage samples of 400-500 g per plot were oven-dried at 103°C for 24 h to determine dry matter content and thus the dry matter yield per m^2^.

### Statistical analysis

Due to different Polyversum treatments, timing of applications and harvests, analyses were done separately within each location. As results from the first two years for Drválovice (Site 3) have been already published (Pisarčik et al., 2019), only the data for years 2019 and 2020 were analysed in the study reported here. The root morphology traits and annual forage yield were analysed by general mixed model (GLM) including effects of treatment, year, block, and treatment × year interaction where plant density was used as a covariate for some analyses. Tap-root diameter was analysed as mean of all individual plants per treatment whereas LRN was averaged only for branch-rooted plants. Data on the proportion of branch-rooted plants and infected plants expressed as percentage were arcsin-transformed, and back-transformed mean values are presented in the results. Plant root disease score calculated at plant level did not meet the assumption of normality (except for Site 3) and was analysed by Kruskal-Wallis ANOVA for treatment and year, followed by multiple comparisons of mean ranks. The compressed stand height values were evaluated by four-way GLM (treatment, year, block, cut) with treatment × year interaction. In all analyses, effect of block was considered as random whereas other factors were considered as fixed. Significant differences between means were reported using the Tukey HSD test at α = 0.05. Correlation between LRN or FRM class and selected root traits at individual plants across sites was quantified using Pearson linear correlation. All these analyses were carried out using the STATISTICA program (StatSoft, Inc, 2012).

## Results

**Figure 1:**
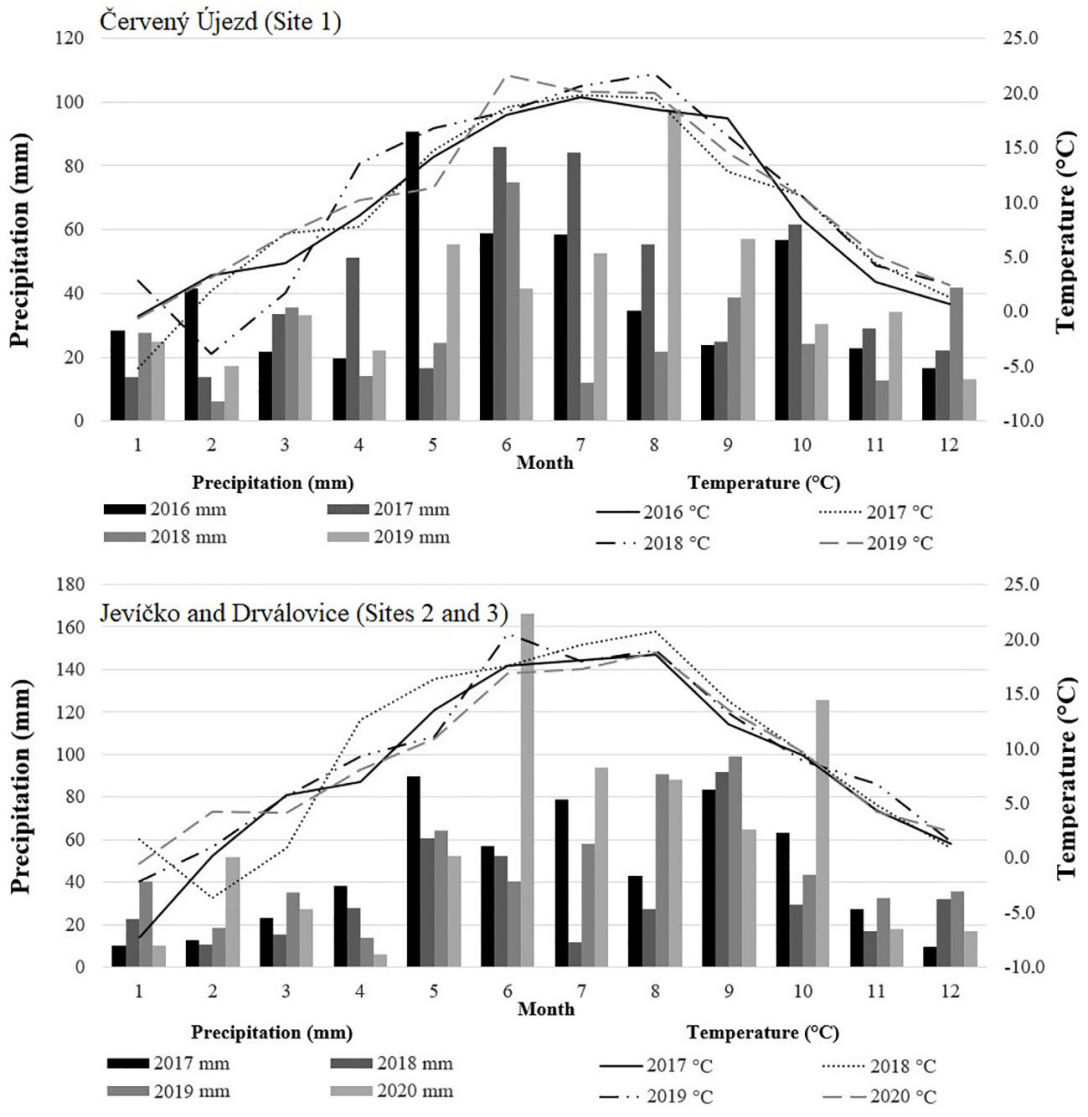
The monthly sums of precipitation and temperature means during the experimental years, from the meteorological station Červený Újezd (Site 1) and Jevíčko (Site 2 and 3), Czech Republic.

Annual temperature mean and sum of precipitation for the experimental locations over the evaluated years are presented in **Table 1**. The monthly precipitation totals and mean temperatures during the growing seasons of 2016 - 2020 are shown in **Figure 1**. At Site 1, all evaluated years were warmer and drier than the long-term mean values for the locality, but the most severe drought stress was observed in 2018 when the growth period from April to October was not only the warmest (16.8°C) but also driest (210 mm). The driest months in this year were April (14 mm) and July (12 mm). For Sites 2 and 3, all evaluated years were also warmer than the long term mean for the locality, with drought stress in 2018. Precipitation was close to the long-term average in 2017 and 2019, with above-average rainfall in 2020.

### Development of root morphology and disease score

For root morphology, a total of 1958 lucerne plants were evaluated, of which 903 were visually detected by root discoloration and 1284 were branch-rooted. The effect of *P. oligandrum* treatment and year on root traits within the locations of the experiments are shown in **Tables 3**–**5**. For Sites 1 and 3, no significant influence of treatments on root traits were observed across the years, but at Site 2 a positive response in the fine root mass was detected for the intensive treatment. At Sites 1 and 2, there was significant year × treatment interaction showing the highest value for plant density in the spring (Site 2) or autumn (Site 3) treatment, relative to the non-treated control, in the seeding year, but there was no impact on forage yield or other root traits, and this effect disappeared in subsequent years (data not shown). For Site 3, the significant interaction showed a higher ratio of branch-rooted plants in the control than in the autumn treatment, but this was associated with higher plant density after Polyversum application in the last year of the experiment.

**Table 3:** Effect of treatment and year for Site 1 (Červený Újezd) on lucerne plant density (PD, plant m^-2^), root potential index (RPI), ratio of infected plants (IP, as %), ratio of branch-rooted plants (RB, as %), total root dry matter (RDM, g m^-2^), tap root diameter (TD, mm), fine root mass score (FRM), plant root disease score of infected plants (PRDS), lateral root number at branch-rooted plants (LRN, pcs plant^-1^), dry matter yield (DMY, t ha^-1^) and compressed stand height (CH, cm).

		PD	RPI	IP	RB	RDM	n	TD	FRM	n	PRDS	n	LRN	n	DMY	CH
	Control	291	84	33.7	59.3	366	16	5.80	3.14	293	2.18	100	2.17	170	12.6^b^	34.9^b^
Treatment	Autumn	334	93	33.1	65.9	391	16	5.96	3.07	309	2.07	102	2.07	191	11.8^a^	33.4^a^
	Intensive	299	90	28.1	63.2	364	16	5.90	2.95	307	2.05	94	2.06	187	11.9^a^	33.4^a^
	*P*	0.476	0.449	0.383	0.628	0.539		0.705	0.107		0.545*		0.759		**0.021**	**<0.001**
	2016	348	44^a^	12.2^a^	62.8^ab^	361	12	3.16^a^	2.76^ab^	149	1.71^a^	14	1.42^a^	84	–	–
	2017	303	97^bc^	39.6^b^	73.7^b^	369	12	6.74^b^	3.18^b^	261	1.74^a^	100	2.65^b^	181	13.4^b^	38.5^c^
Year	2018	296	88^b^	30.8^b^	44.8^a^	374	12	6.33^b^	2.82^ab^	254	2.67^b^	77	2.19^ab^	117	9.6^a^	29.6^a^
	2019	286	114^c^	43.9^b^	67.8^b^	390	12	7.31^d^	3.45^c^	245	2.28^ab^	105	2.15^ab^	166	13.3^b^	33.6^b^
	*P*	0.497	**<0.001**	**<0.001**	**<0.001**	0.228		**<0.001**	**<0.001**		0.006*		**<0.001**		**<0.001**	**<0.001**
Cut		**-**	**-**	**-**	**-**	**-**		**-**	**-**		**-**		**-**		**-**	**<0.001**
Year × treatment	*P*	**0.048**	0.946	0.373	0.360	0.181		0.996	0.071		**-**		0.339		0.522	0.514
Density (covariate)		**-**	**-**	**-**	**0.001**	–		**<0.001**	**0.016**		**-**		**<0.001**		**-**	**-**

P: F - test probability of three-way (year, treatment, block) or four-way (year, treatment, block, cut) GLM including year × treatment interaction (significant values are in bold), different letters indicate statistical differences between treatments or years according to Tukey HSD test at p < 0.05.

* Kruskal - Wallis ANOVA with differences according to multiple comparisons of mean ranks.

**Table 4:** Effect of treatment and year for Site 2 (Jevíčko) on lucerne plant density (PD, plant m^-2^), root potential index (RPI), ratio of infected plants (IP, as %), ratio of branch-rooted plants (RB, as %), total root dry matter (RDM, g m^-2^), tap root diameter (TD, mm), fine root mass score (FRM), plant root disease score of infected plants (PRDS), lateral root number at branch-rooted plants (LRN, pcs plant^-1^), dry matter yield (DMY, t ha^-1^) and compressed stand height (CH, cm).

		PD	RPI	IP	RB	RDM	n	TD	FRM	n	PRDS	n	LRN	n	DMY	CH
	Control	259	81	52.1	70.4	215	12	6.47	3.32^a^	172	2.24	88	2.79	118	10.3	42.0^a^
Treatment	Spring	329	94	51.5	64.6	208	12	6.38	3.60^ab^	224	1.74	109	2.69	140	10.9	42.4^a^
	Intensive	282	89	54.3	67.8	214	12	6.40	3.64^b^	188	1.98	102	2.99	125	11.0	44.7^b^
	*P*	0.170	0.528	0.880	0.994	0.970		0.919	**0.008**		0.105*		0.549		0.366	**<0.001**
Year	2017	441^c^	101	19.3^a^	66.8	263^b^	12	5.73	3.45^b^	188	1.49a	34	3.35	119	–	–
	2018	266^b^	85	65.4^b^	70.0	224^ab^	12	6.09	3.82^c^	234	1.90a	146	2.83	157	11.4^b^	44.7^b^
	2019	163^a^	77	73.2^b^	66.0	148^a^	12	7.44	3.29^a^	162	2.57b	119	2.29	107	10.1^a^	41.4^a^
*P*		**<0.001**	0.108	**<0.001**	0.151	**0.003**		0.205	**<0.001**		<0.001*		0.098		**0.006**	**<0.001**
Cut		**-**	**-**	**-**	**-**	**-**		**-**	**-**		**-**		**-**		**-**	**<0.001**
Year × treatment		**0.043**	0.303	0.694	0.715	0.996		**0.011**	0.942		**-**		0.666		0.866	0.986
Density (covariate)		**-**	–	–	**0.038**	**-**		**0.163**	0.007		**-**		**0.026**			

P: F - test probability of three-way (year, treatment, block) or four-way (year, treatment, block, cut) GLM including year × treatment interaction (significant values are in bold), different letters indicate statistical differences between treatments or years according to Tukey HSD test at p < 0.05.

* Kruskal - Wallis ANOVA with differences according to multiple comparisons of mean ranks.

**Table 5:** Effect of treatment and third and fourth harvest year for Site 3 (Drválovice) on lucerne plant density (PD, plant m^-2^), root potential index (RPI), ratio of infected plants (IP, as %), ratio of branch-rooted plants (RB, as %), total root dry matter (RDM, g m^-2^), tap root diameter (TD, mm), fine root mass score (FRM), plant root disease score of infected plants (PRDS), lateral root number at branch-rooted plants (LRN, pcs plant^-1^), dry matter yield (DMY, t ha^-1^) and compressed stand height (CH, cm).

		PD	RPI	IP	RB	RDM	n	TD	FRM	n	PRDS	n	LRN	n	DMY		CH	
	Control	156	126	73.2	82.1	349	8	10.71	3.25	117	2.26	84	2.71	94	10.5		64.3^a^	
Treatment	Autumn	157	135	65.4	75.2	370	8	10.58	3.08	128	2.71	78	2.63	91	10.4		65.0^b^	
	*P*	0.973	0.509	0.469	0.113	0.608		0.795	0.331		0.051		0.731		0.937		**0.015**	
Year	2019	196^b^	134	67.0	77.6	351	8	10.15	3.30	112	2.05^a^	74	3.08	86	11.1^b^		58.9^a^	
	2020	116^a^	127	71.6	79.7	369	8	10.36	3.03	133	2.91^b^	88	2.05	99	9.8^a^		70.4^b^	
Year		**0.011**	0.589	0.660	0.242	0.685		0.990	0.532		**<0.001**		0.072		**<0.001**		**<0.001**	
Cut		–	–	–	–	–		–	–		–	–	–		–		**<0.001**	
Year × treatment		0.182	0.529	0.196	**0.004**	0.647		0.168	0.265		**-**		0.155		0.855		0.447	
Density (covariate)		**-**	–	–	0.055	–		**<0.001**	0.494		**-**		**0.008**		**-**		**-**	

P: F - test probability of three-way (year, treatment, block) or four-way (year, treatment, block, cut) GLM including year × treatment interaction (significant values are in bold), different letters indicate statistical differences between treatments or years according to Tukey HSD test at p < 0.05.

Effect of year on root traits was generally manifested in a decrease in plant density over time followed by increase of tap-root diameter, root potential index, proportion of infected plants, and plant root disease score along with increasing plant age. Branch-rooted plants percentage and fine root mass varied between years but did not show any consistent trends. In contrast to the general pattern for traits development over years, there were some differences among locations. At Site 1 there was no significant decrease in plant density and this site had the lowest proportion of infected plants (average 30%). For the other two sites, tap root diameter increased over time but changes were not significant. The proportion of infected plants associated with root disease score reached values of around 70% in the last two years.

Results of qPCR detected the pathogens *Fusarium avenaceum*, *F. oxysporum*, and *Verticillium albo-atrum* in all sites and the average percentages of positive detections across sites within each treatment were 39, 19 and 13% for non-treated control, autumn and intensive treatment, respectively.

Relationships between root-branching classes, fine root score, tap root diameter, lateral root number, ratio of infected plants and plant disease score are presented in **Table 6**. Increasing the number of plant lateral roots simultaneously increased tap-root diameter, fine root mass, and proportion of infected plants. Higher fine root score resulted in linear increases in tap-root diameter whereas plant root disease score was improved for plants with fine roots.

**Table 6:** Effect of root branching (RBC) and fine root mass (FRM) class on taproot diameter (TD), fine root mass score (FRM), ratio of infected plants (IP, as %), plant root disease score (PRDS) and lateral root number (LRN) measured as individual plants averaged across all locations and years.

RBC	LRN	TD	FRM	IP	n	PRDS	n	FRM class	TD	IP	n	PRDS	n
0	0	5.87^a^	2.86^a^	37.18^a^	676	2.42^b^	259	1	5.96^a^	46.36	144	3.39^b^	77
1	1 - 2	7.08^b^	3.20^b^	43.53^b^	797	1.90^a^	367	2	6.90^ab^	43.77	336	2.27^a^	150
2	3 - 4	8.63^c^	3.35^b^	49.78^b^	325	2.17^ab^	167	3	7.03^a^	44.86	707	2.04^a^	308
3	≥5	10.91^d^	3.81^c^	63.74^c^	160	2.13^ab^	110	4	7.40^b^	41.59	455	1.94^a^	201
								5	8.52^c^	44.41	316	1.89^a^	167
	*P*	**<0.001**	**<0.001**	**<0.001**		<0.001*			**<0.001**	0.783		<0.001*	
Year	*P*	**<0.001**	**<0.001**	**<0.001**		<0.001*			**<0.001**	**<0.001**		<0.001*	
Linear effects		**0.43**	**0.19**	**0.16**		**0.09**			**0.19**	0.01		**-0.11**	

Linear effects represent Pearson correlation coefficients between LRN or FRM class and root traits with values significant at p < 0.05 in bold.

P: F – test probability; two-way ANOVA (year, branching class); different letters indicate statistical differences between classes.

* Kruskal - Wallis ANOVA with differences according to multiple comparisons of mean ranks.

### Forage yield and canopy traits

In the seeding year, annual forage yield ranged from 2 t ha^-1^ for one-cut regime (at Sites 1 and 3) to 6.4 t ha^-1^ for three-cut regime (at Site 2) although no significant differences between treatments were detected (data not shown). Therefore, **Tables 4**–**6** present only the stand height and yield results from the different harvest years. At Site 1 the forage yield averaged across harvest years was significantly reduced under both of the Polyversum treatments in comparison with the non-treated control. The results for forage yield also corresponded with differences in compressed stand height, with significantly lower values for both Polyversum treatments. In the driest year of the evaluation period (2018) values for forage yield and stand height were lower than in other years. For Sites 2 and 3 no differences in forage yield were detected but the intensive Polyversum treatment at Site 2 and the autumn treatment at Site 3 provided significantly higher values for lucerne stand height.

## Discussion

### Development of root traits in relation to biological control, year, and fine root score

Application of *P. oligandrum*, when applied in both the autumn and intensive schedules in the present study, showed no effects on lucerne root disease score when attacked by *Fusarium* and *Verticillium*. In several other studies, positive responses to *P. oligandrum* in terms of root health of crops were observed (Pharand et al., 2002; Gerbore et al., 2014; Ouhaibi-Ben et al., 2021). Among other root traits, only the fine root-mass score was improved under the intensive treatment at Site 2, Jevíčko. These results are in contrast to those for application of *P. oligandrum* to red clover, where the same treatments were found to support root branching and reduce the plant disease score in the second harvest year (Pisarčik et al., 2020) or they resulted in increased total root dry mass and root potential index across years with drought stress, and with simultaneous improvement of plant disease score in the case of autumn treatment (Pisarčik et al., 2021). The different responses of red clover and lucerne may be explained, at least partly, by differences in the aridity of the climate at the experimental sites. The total annual precipitation for the location of the red clover site of Pisarčik et al. (2021) (at Větrov, Czechia) was always over 530 mm (range 538 - 701 mm), despite some drought periods, and the annual temperature range was 8.2 - 9.4°C. In the present study, the location of Site 1 (Červený Újezd) had annual precipitation below 500 mm in all years of evaluation (334 - 492 mm) with annual mean temperatures in the range 9.5 - 10.4°C. At the more humid location of Jevíčko (Site 2) with alluvial soil and rainfall over 500 mm (except in 2018) there was a positive effect of *P. oligandrum* on the fine-root score under the intensive treatment. In Drválovice (Site 3 in the present study) with loamy-sandy soil, Pisarčik et al. (2019) observed at this same location an increased proportion of infected lucerne plants after spring *P. oligandrum* application, but only for the relatively dry year of 2018 (401 mm and 9.6°C). In two subsequent years rainfall was 574 and 722 mm and no negative effects were observed. These results suggest that responses to *P. oligandrum* application depend on the combined environmental effects of soil and weather, rather than on differences in the responses by lucerne and red clover.

Root development over successive years of the experiments followed general patterns observed previously, including increase in tap-root diameter or root branching with plant age (Lamb et al., 1999). The negative effect of drought on root development was visible especially for Site 1, at the driest location of Červený Újezd, where tap-root increase was strongly reduced with a final value of around 7 mm. This contrasts with the 12 mm reported by Hakl et al. (2017) for comparable plant age at the same location. Effect of the dry year 2018 on tap-root diameter was also obvious at Jevíčko and Drválovice (Sites 2 and 3) (Pisarčik et al., 2019). It demonstrates that the severe drought stress that occurred during this experiment could be considered as a significant factor in not only reducing forage yield and the potential for a biological control effect, but also in terms of its impact on development of root traits and their subsequent agronomic stand performance, in line with Hakl et al. (2021).

The positive impact of higher fine-root score on root disease resistance, as presented in **Table 6**, generally supports a pattern about existing relationships between root traits and disease infection. Similar positive effects of fine-root mass on root disease score were observed for red clover by Pisarčik et al. (2020), and Lamb et al. (2000) reported improved Fusarium wilt resistance for a lucerne population selected for fibrous root mass, together with increased forage yield. According to Hakl et al. (2017), a higher value for lateral root number was positively correlated with higher disease score of lucerne plants, although results from the present study show that this phenomenon is related more to an increasing proportion of infected plants than to a higher root disease score of the infected plants. It seems that the presence of fine roots has a positive role here, which was associated mainly with improved disease score of infected plants. However, it should be remembered that although this trait could be effective for selection (Lamb et al., 2000), fine root mass and lateral root number are correlated with each other (Lamb et al., 1999) and there are also a number of seasonal patterns affecting the presence of fine roots, such as soil temperature and carbohydrate supply (Luo et al., 1995).

### Effect of *Pythium oligandrum* on forage yield in association with root morphology

Across the three experiments with lucerne reported here, our results showed that application of *P. oligandrum* did not provide a positive yield response, and that at the warmest and driest location the forage yield was even slightly reduced (by 6%) together with reduced stand height. In more humid environments, a positive effect of either the intensive or autumn application on stand height was observed but this advantage was not realised in any significant forage yield improvement. The positive effect is consistent with the beneficial effects of root colonization by *P. oligandrum* on crop growth promotion as described by Benhamou et al. (2012) or Gerbore et al. (2014). In line with Pisarčik et al. (2021), the greater stand height could not be attributed fully to the improved root traits at Site 2 (Jevíčko), because greater stand height was observed also at Site 3 (Drválovice) where there was no effect on roots. We may conclude, therefore, that the observed positive effect of *P. oligandrum* on lucerne forage growth was not related to root traits. The efficacy of *P. oligandrum* was driven by environmental conditions, in which colder and more humid conditions promote a positive response when recorded in the field. This further emphasises the importance of temperature and other aspects of weather during the growing season on the activity and efficiency of *P. oligandrum* (Boček et al., 2012; Baturo-Cieśniewska et al., 2018).

Apart from the effect of humidity on *P. oligandrum* activity, some negative effects of biological control on crop biomass accumulation in early stages of growth have been reported (McGehee et al., 2019), in which there was a negative effect on crop roots and aboveground biomass especially when the pathogen was not inoculated along with biological control. This could also be relevant in the present study, where there was a negative effect of *P. oligandrum* at the location with the lowest ratio of infected plants, whereas some positive effects (on fine root mass score, stand height) were observed in both locations with the proportion of infected plants around 70%. In previous work, a positive response of red clover was also reported under high pressure of root diseases, where about 90% of plants were positively scored for root discoloration in the last year of the field experiments (2021; Pisarčik et al., 2020). However, the effect of higher plant infection cannot be easily separated from the temperature-precipitation relationship. Nevertheless, we can speculate that reduced attack by soil pathogens could also have some association with the observed negative effects on lucerne stand height and yield performance.

Results of two independent field experiments with red clover suggest that the stimulation effect of *P. oligandrum* on stand height played a more important role in increased vegetative biomass yield than through a direct protection against fungal diseases or root traits improvement (2021; Pisarčik et al., 2020). The results for lucerne reported here are in line with those previous findings, as autumn or spring + autumn applications of *P. oligandrum* per year were associated with increased stand height without any response in the ratio of infected plants and root disease score. However, either a single autumn, or two applications, were not sufficient for significant yield enhancement, and the single spring application did not provide any significant effect, in line with the suggestion of Pisarčik et al. (2021) about the need to optimize the application timing over the second half of the growing season. An important result here could be a negative lucerne yield response on this potential stimulation under the relatively dry environment. Pisarčik et al. (2021) considered that the stimulation of red clover is related to the ability of *P. oligandrum* to synthesize tryptamine in direct interaction with plant roots, and root absorption of this newly formed auxin-compound in appropriate concentrations has been associated with enhancement of plant growth, in line with Le Floch et al. (2003) or Binyamin et al. (2019). Although auxins stimulate plant growth and also could be beneficial for plant defence to drought stress (Zhang et al., 2012; Liu et al., 2014; Korver et al., 2018) it must be remembered that sustaining growth under unfavourable conditions could be detrimental (Dubois et al., 2018). *Pythium oligandrum* also activates the plant defence system through production of oligandrin and the cell-wall protein fraction which appear to be closely involved in activation of the jasmonic acid and ethylene dependent signalling pathways (Benhamou et al., 2012). Ethylene has an important role in regulation of organ growth and yield under abiotic stress (Dubois et al., 2018) therefore its imbalance could be also responsible for reduced lucerne growth and yield. This field-based study clearly demonstrated that application of *P. oligandrum* under drought stress did not support lucerne root development and it may even affect forage production negatively, probably due to the imbalance between auxins and ethylene.

These relationships between the legume plant and *P. oligandrum* can also be largely influenced by the total plant microbiome, where plant–microbiome interactions are significant determinant for plant growth, fitness and productivity (Gupta et al., 2021). Understanding the plant microbiome interactions within the microbial community can contribute to hypotheses about why biological control is inconsistent in promoting or reducing crop growth (Berendsen et al., 2012) Understanding the mechanisms linked to the positive effects of beneficial fungi is also essential for achieving favourable outcomes and development of novel strategies (Ghorbanpour et al., 2018). In field conditions, these relationships are further complicated by interactions with environment, where results of biological control of crop diseases are more variable in comparison with the controlled conditions of glasshouse or laboratory studies (Boček et al., 2012; Pisarčik et al., 2019).

It can be summarized that under the relatively dry climate with mean temperature close 10°C and annual total precipitation below 500 mm, there was reduced potential of *P. oligandrum* for improvement of lucerne root traits, disease score or forage yield and where even a negative effect on crop growth has been observed. This negative response could be probably associated with inappropriate stimulation or disturbance of the balance between auxins and ethylene, and which seems to be mitigated on deep, more humid soils. Under conditions with annual precipitation over 500 mm, the positive impact on fine root mass score and stand height was observed for the autumn and intensive treatments but this advantage was not realised in significantly increased yield. Therefore, we conclude that although there is some potential for *P. oligandrum* for lucerne productivity improvement, this is most likely to be realised only under conditions of relatively humid environments or under irrigation, where higher root disease infestation by *Fusarium* or *Verticilium* may also be expected, and as found previously for red clover, more applications per year will be needed for forage yield improvement. Further research is also needed in plant-microbiome interactions in association with biological control agents which could be essential for development of effective strategies of crop biological control.

## Data availability statement

The raw data supporting the conclusions of this article will be made available by the authors, without undue reservation.

## Author contributions

MP: writing-original draft, data analysis, preparation, writing-review and editing. JH: conceptualization, investigation, writing-original draft, supervising, editing OS: investigation, writing-review PN: editing. All authors contributed to the article and approved the submitted version.
